# DNA secondary structure formation by DNA shuffling of the conserved domains of the Cry protein of *Bacillus thuringiensis*

**DOI:** 10.1186/s13628-017-0036-7

**Published:** 2017-05-22

**Authors:** Efrain H. Pinzon, Daniel A. Sierra, Miguel O. Suarez, Sergio Orduz, Alvaro M. Florez

**Affiliations:** 1 Laboratory of Biotechnology and Molecular Biology, MASIRA Institute, School of Health, University of Santander, UDES, Bucaramanga, Colombia; 2 School of Electrical, Electronics and Telecommunications Engineering, Universidad Industrial de Santander, Bucaramanga, Colombia; 3 School of Biociencies, Faculty of Science, National University of Colombia, Medellin campus, Medellin, Colombia; 4 Present address: RG Microbial Ecology: Metabolism, Genomics & Evolution | Div. Ecogenomics & Holobionts, Microbiomas Foundation, CL 19 5A 64 CS 46, 250001 Chia, Colombia

**Keywords:** *Bacillus thuringiensis*, Cry toxins, DNA shuffling, DNA secondary structures, *in silico* modeling

## Abstract

**Background:**

The Cry toxins, or δ-endotoxins, are a diverse group of proteins produced by *Bacillus thuringiensis*. While DNA secondary structures are biologically relevant, it is unknown if such structures are formed in regions encoding conserved domains of Cry toxins under shuffling conditions. We analyzed 5 holotypes that encode Cry toxins and that grouped into 4 clusters according to their phylogenetic closeness. The mean number of DNA secondary structures that formed and the mean Gibbs free energy $\left(\overline{\varDelta G}\right)$ were determined by an *in silico* analysis using different experimental DNA shuffling scenarios. In terms of spontaneity, shuffling efficiency was directly proportional to the formation of secondary structures but inversely proportional to ∆G.

**Results:**

The results showed a shared thermodynamic pattern for each cluster and relationships among sequences that are phylogenetically close at the protein level. The regions of the *cry11Aa, Ba* and *Bb* genes that encode domain I showed more spontaneity and thus a greater tendency to form secondary structures (<∆G). In the region of domain III; this tendency was lower (>∆G) in the *cry11Ba* and *Bb* genes. Proteins that are phylogenetically closer to Cry11Ba and Cry11Bb, such as Cry2Aa and Cry18Aa, maintained the same thermodynamic pattern. More distant proteins, such as Cry1Aa, Cry1Ab, Cry30Aa and Cry30Ca, featured different thermodynamic patterns in their DNA.

**Conclusion:**

These results suggest the presence of thermodynamic variations associated to the formation of secondary structures and an evolutionary relationship with regions that encode highly conserved domains in Cry proteins. The findings of this study may have a role in the *in silico* design of *cry* gene assembly by DNA shuffling techniques.

## Background

DNA secondary structures have key biological roles, as they are involved in processes such as DNA replication, transcription, recombination and repair [1–3]. Thus, single-strand DNA secondary structures contain information, similarly to double-stranded DNA, in their nucleotide sequences. When using molecular techniques, the formation of secondary structures can affect hybridization, leading to false positives and cross-reactions [4–6]. In techniques such as DNA shuffling [7], where successive cycles of DNA amplification are performed with and without primers, secondary structures can be generated that alter genetic variability during recombination.

In *in silico* models of DNA shuffling, the role of secondary structure formation during DNA shuffling has not been studied. Among the *in silico* models available, some have focused on simulation and prediction [8, 9], while others have focused on the integration of kinetic elements in a Markov model [10] and on the optimization of the DNA shuffling reaction [11]. These tools have used a Poisson-exponential distribution [12] to simulate DNA fragmentation and have employed the unified calculation of free energy using the nearest-neighbor described by SantaLucia (1998) [13]. This calculation has also been used as a thermodynamic parameter to predict the formation of secondary structures in computational tools such as UNAFold, *Unified Nucleic Acid Folding* [14] and NASP, *Nucleic Acid Secondary Structure Predictor* [15]. Both tools calculate Gibbs free energy and Boltzmann’s probability [16]. NASP has additional elements that calculate the conservation level of structures and thermodynamic stability [15]. Similarly, these tools are supported by dynamic programming algorithms and use databases that contain thermodynamic parameters that are supported by servers such as DNA-MFOLD [17], OMP (*Oligonucleotide Modeling Platform*; DNA Software Inc.) [18] and NASP [15]. These tools have demonstrated their utility for the prediction of secondary structures, and they were recently used to design DNA barcodes in plants based on RNA ITS/ITS2 transcripts [19] and to elucidate the roles and biological significance of secondary structures in some viruses [1].

The Cry toxins, or δ-endotoxins, constitute a group of 74 toxins and 295 holotypes [20] that belong to the 3 domain (3D) family of proteins produced by *Bacillus thuringiensis* (*Bt*). These toxins have been used in agronomical pest control for decades showing conserved amino acid blocks and variable specificity to different insect orders [21, 22]; they cause insect death via the formation of membrane pores or by forming ion channels [23]. The 3 domains are associated with different aspects of the toxic mechanism. Domain I is involved in pore formation, domain II is involved in toxin specificity and in binding to the epithelial receptors of the midgut in insects, and domain III, which is least characterized, has been suggested to stabilize the toxin-receptor interaction that leads to osmotic imbalance and thus to insect death [23, 24]. Each of the 3 domains makes an individual contribution to insect specificity, and they show correlations between sequence similarity and specificity, even between phylogenetically distant groups with similar activities. Specificity may have developed along multiple evolutionary paths; the relative similarity between regions and domains suggests co-evolution, and the differences found in domain topology suggest that positive pressure on domains II and III and swapping of domain III sequences may represent ways to promote Cry toxin diversity [25, 26]. Furthermore, the existence of certain genetic patterns in native Cry toxins that increase toxicity and promote diversity has been suggested [26]. Currently, there is scientific evidence for the specificities of these proteins related to their sequences, and different molecular approaches have been not only used to an improved understanding of the mode of action, but also to creating stable and functional proteins with increased toxic activity [25–27].

In order to know whether the formation of secondary structures has an effect on Cry toxins under DNA shuffling conditions, we propose to predict changes in secondary structures in terms of the efficiency using Computer-Assisted Mutagenesis (CAM) tool. This study shows a different way to study the thermodynamic behavior of *cry* genes associated with the formation of DNA secondary structures under the experimental conditions of DNA shuffling*.* Our findings elucidate a thermodynamic behavior pattern that is associated with the phylogeny among studied genes, and they thus represent an input of interest in the design of DNA shuffling experiments.

## Methods

**Fig. 1:**
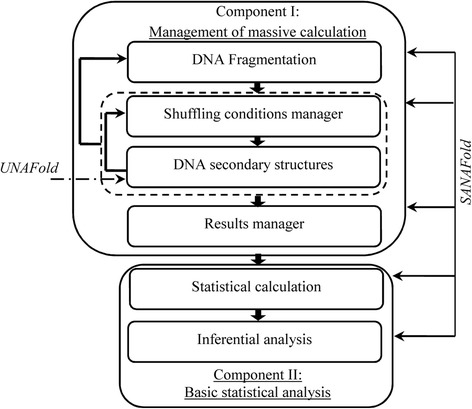
Implementation based on two computational components of the *in silico* strategy. The SANAFold software architecture consists of two components as follow: component I that manages the simulation scenarios and the massive calculation of thermodynamic values associated with DNA secondary structures in those scenarios and component II that enables basic statistical analysis

We designed a software program termed Statistical Analysis of Nucleic Acid Folding (SANAFold), which was written in Python language and executed in Beowulf Cluster architecture with Linux (Distribution Fedora 20). The software was divided into 2 functional components. The first component included DNA sequence fragmentation, management of the massive calculation of DNA secondary structure and simulation scenarios. For the massive calculation, the thermodynamic calculations for simulations of secondary structures employed UNAFold software [14]. The second component included the statistical analysis that allowed inferences to be drawn from the data obtained by the first component (Fig. 1).

### Fragmentation of DNA sequences

DNA fragmentation was performed with 4 clusters of cry genes (I, II, III and IV) that were grouped by their phylogenic closeness (Table 1). For each of the sequences, the gene regions encoding the 3 domains described for Cry toxins [24] were identified and then fragmented to simulate DNase I digestion, which is used for DNA shuffling experiments [7, 12]. The gene sequences were entered as multifasta files. The fragmentation of the domains used a random selection of cut sites from a cumulative Poisson distribution where:

**Table 1:** Clusters of studied *cry* genes

Cluster	Toxin	Reference	Source	Open Reading Frame		
				AA	St - Sp (bp)	# GenBank Access
I	Cry11Aa1	Donovan et al. 1988 [32]	*Bt israelensis*	646	32-1972	M31737- J03510
	Cry11Ba1	Delecluse et al. 1995 [33]	*Bt jegathesan*	724	64-2238	X86902
	Cry11Bb1	Orduz et al. 1998 [29]	*Bt medellin*	786	1-2346	AF017416
II	Cry2Aa1	Donovan et al. 1988 [32]	*Bt kurstaki*	633	156-2057	M31738.1
	Cry18Aa1	Zhang et al. 1997 [34]	*Paenibacillus popilliae* ^*a*^	712	725-2863	X99049
III	Cry1Aa1	Schnepf et al. 1985 [35]	*Bt kurstaki* HD 1	1176	527-4057	M11250.1
	Cry1Ab1	Wabiko et al. 1986 [36]	*Bt berliner 1715*	1155	1-1695	M13898.1
IV	Cry30Aa1	Juarez-Perez et al. 2003 [37]	*Bt medellin*	662	60-2045	AB125059
	Cry30Ca1	Sun et al. 2013 [38]	*Bt jegathesan*	688	1-2064	GQ368655

^a^ *Firmicutes* bacterial phylum, *Bacilli* class, *Bacillales* order, *Paenibacillaceae* family, *Paenibacillus* genus. The conformation of the four study clusters is grouped under the criterion of evolutive closeness

1 $$ {f}_x\left( X\le x\right)=1 - {e}^{-\lambda x} $$

where λ = 1/l is a parameter that indicates the number of n success of cuts in the sequence, l is the length of the DNA fragments within a range of 50 and 250 bp and, x is a random variable between 0 and 1 that is produced to find the cuts lengths.

The simulations were performed in SANAFold and included 5 replicates of fragmentation, an artifice that allowed us to enter the desired statistical variation. This software did not store the fragments for analysis but it makes n replicates that in our case were 5. The fragments resulting from the fragmentation process were organized as one-dimensional arrays F [i] where i is the number of fragments obtained in each fragmentation replicate.

In order to obtain thermodynamic data, the one-dimensional arrays F [i] were processed by UNAFold 3.8 according to folding parameters with a sodium concentration of 0 mM.

The gene sequences were entered as multifasta files, and the fragments resulting from the fragmentation process were organized as one-dimensional arrays *F*[*i*] where *i* is the number of fragments obtained in each fragmentation replicate.

### Simulation scenarios

Each simulation scenario consisted of subjecting the DNA sequences to 3 experimental DNA shuffling conditions to determine the formation of DNA secondary structures. The 3 experimental conditions (variables) were temperature (TE), Mg^++^ concentration (MA) and mean expected fragment length from the nucleotide sequence (LE). To develop simulations and analyze the experimental conditions, *in silico* experiments allowing variations in the experimental conditions were designed in groups of 2, leaving the third condition as a constant (parameter). Each DNA fragment generated during the previous step was evaluated while considering ranges of values for the 3 experimental conditions, namely TE = 48 °C - 68 °C, MA = 0.02 mM - 1 mM and LE = 50–250 bp. The mean values for the established ranges were TE = 48 °C, MA = 0.5 mM and LE = 50 bp. These values were considered as parameters for the simulation scenarios; thus, combinations of mean fragment length values and temperatures were performed in LE- TE scenarios, keeping MA constant at 0.5 mM. In LE-MA scenarios, combinations of mean fragment length values and the ionic magnesium concentration were tested, keeping the TE constant at 48 °C. Combinations of temperatures and ionic magnesium concentrations were also tested while keeping LE constant at 150 bp. A reference scenario was also established in which all genes were assessed by clusters under LE-MA conditions with a range of LE values = [50–250 bp.], TE = 37 °C and MA = 0.02 mM. The variables and parameters used for the simulation scenarios were taken from previous experimental DNA shuffling conditions [Unpublished observations, Florez AM, Suarez-Barrera MO, Morales GM, Rivera KV, Orduz S, Ochoa R, Guerra D, Muskus C] and were used to compare the *in silico* results for Cry11 toxins.

### Simulation of DNA secondary structure

The massive calculation management component was performed under the different simulation scenarios detailed above. The formation of DNA secondary structures was determined by executing the software UNAFold, after preparation of the inputs to diverse scenarios, for each DNA fragment generated and with combinations of simulation scenarios [14]. The results generated by UNAFold were sent to the massive calculation management component with the thermodynamic information for each of the possible DNA secondary structures, which were subsequently stored in a consolidated file with the extension*.csv* (comma-separated values), in which approximately 18.2*10^6^ thermodynamic data points were stored (Fig. 1).

The spontaneity criterion was related to the thermodynamic capacity of a gene region to favor the formation of DNA secondary structures. In this case, if the mean ∆G of a DNA secondary structure of one gene region is more negative than another, it is considered that the former is more spontaneous, that is, it has a greater tendency to form DNA secondary structures compared to other regions of the same gene. Therefore, it was assumed in this study that the degree of spontaneity (high, medium or low) is a useful measure resulting from the qualitative assessment of the dispersion of datasets for each *cry* gene that showed *f-ratio* values with significant differences.

### Statistical analysis

The simulation data were replicated 5 times and were consolidated in 2-dimensional arrays in*.csv* files with 3 variables of interest: i) the mean number of secondary structures formed by a DNA fragment; ii) the *Δ*G values of the predicted DNA secondary structures; and iii) the percentage of DNA secondary structures obtained with *Δ*G (-). The obtained data were grouped into results with *ΔG* (-) and *Δ*G (+), thereby yielding 6 statistical estimators from the 3 variables of interest. Statistical calculations were performed by analysis of variance (ANOVA) using 3 datasets assessing one factor. The datasets belonged to each of the 6 established estimators, and the factor was related to the biological origin of the data; thus, data from the 3 previously fragmented gene regions encoding each domain of the same protein were subjected to the ANOVA analysis. Values of *f-ratio* were obtained to evaluate the existence of significant differences between the means of the variants. From data with significant differences, a qualitative analysis of data dispersion was performed by analyzing box-whisker diagrams plotted by SANAFold. A significant difference in the *f-ratio* value indicates that the degree of spontaneity of at least one of the means can be distinguished from the others. This differentiated mean corresponds to a low or high spontaneity depending on whether it is qualitatively located at one of the extremes relative to the means of the remaining gene regions. The ANOVA allowed the identification of *f-ratio* values with their respective degrees of freedom and a confidence interval of 95.5%, which exceeded the threshold for acceptance of the null hypothesis of a Fisher’s distribution, where the null hypothesis (Ho) was assumed as the equality of means in the analyzed data.

The measure of spontaneity was assessed from the behavior of estimator data that showed *f-ratio* values that exceeded the threshold for acceptance of Ho. The established estimators were mean $\overline{\varDelta \mathrm{G}}$ of the DNA secondary structures with *Δ*G (-) and *Δ*G (+), the mean number of DNA secondary structures formed from fragments of *cry* gene sequences with *Δ*G (-) and *Δ*G (+), and, finally, the percentage of DNA secondary structures with *Δ*G(‐) and *Δ*G(+).

## Results

**Fig. 2:**
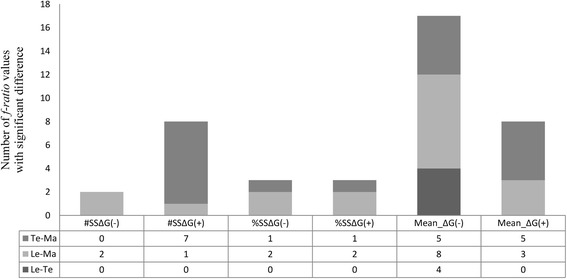
Behavior of statistical estimators in *cry* gene clusters. #SSΔG = number of secondary structure with ΔG (*negative or positive*); %SSΔG = percentage of secondary structure with ΔG (*negative or positive*); Mean_ ΔG = Mean of the free energy (*negative or positive*). TE-MA: scenario conformed by variations of Temperature-Magnesium. Le-Ma: scenario conformed by variations of Length-Magnesium. Le-Te: scenario conformed by variations of Length-Magnesium

A total of 162 *f-ratio* values were obtained as a product of the statistical analysis that summarized the thermodynamic behavior of *cry* gene clusters. Among them, 41 *f-ratio* values, representing 25.3% of the analysis and slightly more than 4.6 * 10^6^ of the calculated thermodynamic data points, showed statistically significant differences (Table 2). Among the statistically significant *f-ratio* values, the behavior of the data was reviewed with the respective statistical estimator, which allowed us to detect tendencies in the prevalence of regions that favor or disfavor the formation of DNA secondary structures. Based on the review, the estimator $\overline{\Delta \mathrm{G}\left(-\right)}$ was the most representative out of the 6 estimators used in this study, with a presence of 41.4%. Thus, the 4 clusters were assessed with the estimator $\overline{\Delta \mathrm{G}\left(-\right)}$. When they were subjected to simulated scenarios with different experimental DNA shuffling conditions, they revealed distinct thermodynamic activities among gene regions that encode for each domain that favored the formation of DNA secondary structures (Fig. 2). Furthermore, the greatest frequencies of variation in estimators with enough significant differences in the formation of DNA structures among regions were found with a combination of parameters that included variation of Mg^++^, with 18 significant *f-ratio* values in LE-MA conditions and 19 significant *f-ratio* values in TE-MA conditions. In LE-TE conditions, that is, in the absence of Mg^++^ variations, only 4 *f-ratio* values showed significant differences (Fig. 2).

**Table 2:** *F-ratio* values with statistically significant differences (regions of each gene *cry11* of *Bacillus thuringiensis*)

Cluster	DNA	*f-ratio*						DNA shuffling conditions	DF (n:d)^c^	*f-ratio* measurement
		# SSΔG^a^		% SSΔG^b^		ΔG				
		-	+	-	+	-				
I	*cry11Aa1*							LE-TE	2:24	3.40
						6.60		LE-MA	2:33	3.28
			5.97			8.25	3.30	TE-MA	2:33	3.28
	*cry11Ba1*							LE-TE	2:24	3.40
		6.45		4.41	4.41	9.90	3.30	LE-MA	2:33	3.28
			8.69				3.30	TE-MA	2:33	3.28
	*cry11Bb1*							LE-TE	2:24	3.40
							4.12	LE-MA	2:33	3.28
			8.81			4.12		TE-MA	2:33	3.28
II	*cry2Aa1*					9.00		LE-TE	2:24	3.40
						28.00		LE-MA	2:33	3.28
						6.19	4.12	TE-MA	2:33	3.28
	*cry18Aa1*							LE-TE	2:24	3.40
					5.50			LE-MA	2:33	3.28
								TE-MA	2:33	3.28
III	*cry1Aa1*					4.26		LE-TE	2:24	3.40
						21.35		LE-MA	2:33	3.28
			6.85	5.44	5.73	7.50	4.71	TE-MA	2:33	3.28
	*cry1Ab1*					3.60		LE-TE	2:24	3.40
						16.50	3.30	LE-MA	2:33	3.28
			5.77					TE-MA	2:33	3.28
IV	*cry30Aa1*					6.00		LE-TE	2:24	3.40
		4.69				8.25		LE-MA	2:33	3.28
			6.67					TE-MA	2:33	3.28
	*cry30Cal*							LE-TE	2:24	3.40
			5.22	4.58	4.67	7.62		LE-MA	2:33	3.28
			8.21			4.12	7.07	TE-MA	2:33	3.28

^a^#SSΔG = number of secondary structure with ΔG (negative or positive); ^b^%SSΔG = percentage of secondary structure with ΔG (negative or positive); ^c^ *DF* Degrees of Freedom, *n* numerator, *d* denominator

### Reference scenario

**Fig. 3:**
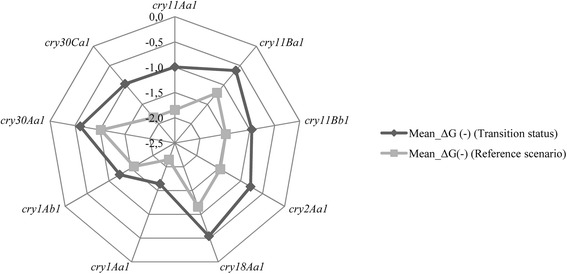
Thermodynamic (kcal/mol) comparison between the reference scenario and the transition status in simulated LE-MA DNA shuffling conditions of *cry* genes from *Bacillus thuringiensis.* Reference scenario are the average of the free energies of the DNA secondary structures obtained from one scenario. LE-MA conditions with a range of LE values = [50–250 bp.], TE = 37 °C and MA = 0.02 mM. Transition status are the average of the free energy of the DNA secondary structures obtained in scenario LE-MA

In the simulated reference scenario, the *cry* gene clusters showed favorable behavior for the spontaneous formation of DNA secondary structures. Energy ranges from the estimator $\overline{\Delta \mathrm{G}\left(-\right)}$, which establish the mean energies of DNA secondary structures formed by fragments of *cry* genes, varied between -1.0 and -2.2 kcal/mol. The genes showing the greatest spontaneity were *cry1Aa, cry11Aa* and *cry30Ca* (Fig. 3). In general, *cry* clusters showed a decrease in their thermodynamic capacity to spontaneously form DNA secondary structures in the experimental DNA shuffling conditions LE-MA when compared with the reference scenario. This result was evidenced by the less-negative values of the $\overline{\Delta \mathrm{G}\left(-\right)}$ estimator (Fig. 3).

### First cluster analysis

**Fig. 4:**
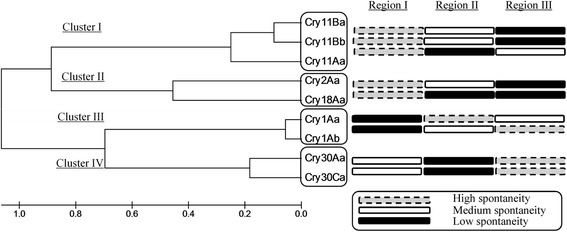
Association of the thermodynamic behavior of DNA secondary structure formation in regions of *cry* genes with their phylogenetic clustering. Thermodynamic spontaneity of the gene regions from *cry* gene coding sequences, are shown by cluster of Cry proteins according to their evolutive proximity

Analysis cluster I corresponded to the genes *cry11Aa1, cry11Ba1* and *cry11Bb1*. For this cluster, 54 *f-ratio* values were assessed, and 14, equivalent to 25.9% of the analysis, showed significant differences (Table 2). When assessing data dispersion between the regions of the *cry11Aa1* gene that encode domains I, II and III, 4 *f-ratio* values with significant differences were found under simulation conditions of LE-MA and TE-MA. In all cases, analysis of the dataset for each *f-ratio* showed the same thermodynamic behavior by region, namely, high spontaneity in the gene region that encodes domain I, moderate spontaneity in the region that encodes domain III and low spontaneity in the gene region that encodes domain II (Fig. 4).

Seven *f-ratio* values with statistically significant differences were found under simulation conditions LE-MA and TE-MA (Table 2) when evaluating data dispersion between the regions of the *cry11Ba1* gene that encode domains I, II and III. In all cases, the dataset for each *f-ratio* showed the same thermodynamic behavior by region, namely, high spontaneity in the gene region that encodes domain I, moderate spontaneity in the gene region that encodes domain II and low spontaneity in the gene region that encodes domain III (Fig. 4). Finally, 3 *f-ratio* values with statistically significant differences were found under simulation conditions LE-MA and TE-MA (Table 2) when assessing the regions of the *cry11Bb1* gene that encode domains I, II and III. Furthermore, the thermodynamic behavior of the dataset for each *f-ratio* by region was similar, showing the same result in terms of spontaneity as the *cry11Ba1* gene (Fig. 4). No significant differences were found in this cluster under the LE-TE simulation conditions.

### Second cluster analysis

Analysis of cluster II corresponded to the *cry2Aa1* and *cry18Aa1* genes. For this cluster, 36 *f-ratio* values were evaluated, 5 of which (13.8% of the analysis) showed statistically significant differences (Table 2). When assessing the dataset dispersion between the regions of the *cry2Aa1* gene that encode domains I, II and III, 4 *f-ratio* values with significant differences were found under the LE-MA, LE-TE and TE-MA simulation conditions (Table 2). In these cases, the thermodynamic behavior by region showed the same result in terms of spontaneity as the *cry11Ba1* and *cry11Bb1* genes (Fig. 4). Finally, 1 *f-ratio* value with a significant difference in LE-MA conditions was found for this cluster when assessing the regions of the *cry18Aa1* gene that encode domains I, II and III (Table 2). The tendency of the thermodynamic behavior showed the same result as obtained with the *cry11Ba1* and *cry11Bb1* genes for regions I and III (Fig. 4).

### Third cluster analysis

Analysis of cluster III corresponded to the *cry1Aa1* and *cry1Ab1* genes. For this cluster, 36 *f-ratio* values were assessed, 11 of which (30.5% of the analysis) showed significant differences (Table 2). Seven *f-ratio* values with significant differences under all shuffling conditions were found when reviewing the dispersion of data between the regions of the *cry1Aa1* gene that encode domains I, II and III. A larger variation in estimators was found when the gene sequences were assessed under the TE-MA simulation conditions (Table 2). In all cases, analysis of the dataset of each *f-ratio* showed the same thermodynamic behavior by region, with high spontaneity in the gene region that encodes domain II, moderate spontaneity in the gene region that encodes domain III and low spontaneity in the gene region that encodes domain I (Fig. 4).

Finally, the regions of the *cry1Ab1* gene that encode domains I, II and III showed 4 *f-ratio* values with significant differences under the LE-TE, LE-MA and TE-MA simulation conditions. Analysis of the dataset of each *f-ratio* revealed results that differed from those for *cry1Aa1*, as they suggested high spontaneity in the gene region that encodes domain III, moderate spontaneity in the gene region that encodes domain II and low spontaneity in the gene region that encodes domain I (Fig. 4).

### Fourth cluster analysis

Analysis of cluster IV corresponded to the *cry30Aa1* and *cry30Ca1* genes. For this cluster, 36 *f-ratio* values were assessed, 11 of which (30.5% of the analysis) showed significant differences (Table 2). The data dispersion between the regions of the *cry30Aa1* gene that encode domains I, II and III showed 4 *f-ratio* values with significant differences under the LE-TE, LE-MA and TE-MA simulation conditions (Table 2). Analysis of the dataset of each *f-ratio* showed the same thermodynamic behavior, namely, high spontaneity in the gene region that encodes domain III but variations between moderate and low spontaneity in the gene regions encoding domains I and II, with the estimator $\overline{\varDelta G\left(-\right)}$ showing a prevalence for moderate spontaneity in the region that encodes domain I and a prevalence for low spontaneity in the region encoding domain II (Fig. 4). Conversely, the dispersion of data between the regions of the *cry30Ca1* gene that encode domains I, II and III showed 7 *f-ratio* values with significant differences under the LE-MA and TE-MA simulation conditions. For this gene, the tendency of the thermodynamic behavior by region was maintained, showing high spontaneity in the gene region that encodes domain III, moderate spontaneity in the gene region encoding domain I and low spontaneity in the gene region encoding domain II (Fig. 4).

## Discussion

The biological significance of DNA secondary structures has been described for several cellular events in eukaryotes, prokaryotes and viruses [1–3]. Furthermore, the relevance of DNA secondary structures has been described in molecular biology and biotechnology applications associated with techniques that employ denaturation of DNA strands, where structure formation can lead to inhibition of hybridization or cross-reactivity [28]. In techniques such as DNA shuffling, recombination is the basis for performing assembly of parental genes and promoting genetic variability, and the formation of DNA secondary structures has a key role during recombination. In this study, thermodynamic variations associated with the formation of DNA secondary structures under DNA shuffling conditions in a group of genes belonging to the *cry* family of *B. thuringiensis* was organized into 4 clusters according to their phylogenetic relationship. The gene regions encoding the three (3) highly conserved domains related to Cry toxin function were analyzed.

The thermodynamic behavior of the *cry* genes was similar in all clusters during simulations in the reference scenario. Although no significant thermodynamic variations by region were shown, the genes showed more spontaneity [<∆G(-)] in the reference scenario than under *the* DNA shuffling conditions (Fig. 4). In the LE-MA, TE-MA and LE-TE simulation scenarios, the thermodynamic behavior was similar among *cry* genes that belonged to the same cluster, suggesting that variations in fragment length, temperature and Mg^++^ concentration were decisive in the shuffling conditions. According to the analyses of the spontaneity of gene regions, a thermodynamic pattern could be inferred for each gene cluster.

The first thermodynamic pattern in cluster I (*cry11Aa1, cry11Ba1, cry11Bb1*) showed a greater tendency of the gene region encoding domain I to form secondary structures, due to its high spontaneity and hence its [< ΔG(−)]. However, the thermodynamic pattern in *cry11Aa* was different with respect to the regions that encode domains II and III. Accounting for the thermodynamic behavior of the cluster and the regions, the domain III-encoding region of *cry11Ba1* and *cry11Bb1* showed a lower tendency to form secondary structures than the domain II-encoding region, while the domain I-encoding region showed higher spontaneity. Interestingly, this thermodynamic behavior is maintained between toxins that are most closely related phylogenetically, such as the Cry11Ba1 and Cry11Bb1 toxins (Fig. 4), and that have a greater percentage of identity at the DNA sequence level. Whereas *cry11Ba1* and *cry11Bb1* share 83% identity, they share 62 and 60%, respectively, identity with *cry11Aa1* [29]. This finding could explain the thermodynamic differences found among the genes of a single holotype, Cry11. The conformations of these thermodynamic patterns under conditions of DNA shuffling could suggest a propensity of certain regions to recombine among each other, which would favor genetic variability. Such recombination could have a direct relationship with the results obtained from DNA shuffling experiments performed by our group using the 3 *cry11* genes. It has been found that 48.5% of assembled fragments are mainly incomplete genes that contain domain III with homology to *cry11B* 1and that the 31.4% that manage to assemble complete genes correspond to proteins with the toxic activity of Cry11Aa1 but that showed greater genetic variability as deletions, insertions and substitutions in domain III relative to the regions that encode the other domains [Unpublished observations, Florez AM, Suarez-Barrera MO, Morales GM, Rivera KV, Orduz S, Ochoa R, Guerra D, Muskus C]. The thermodynamic variations obtained at the DNA level were similar among genes that are more phylogenetically related. To determine if such behavior also appeared in genes related to the holotype Cry11, the same assays were performed with the gene sequences of *cry2Aa* and *cry18Aa*, which belong to cluster II. These 2 genes encode 2 toxins that are phylogenetically closer to cluster I [20], with 51.3% identity; the divergence of the domain II structures confers specificity to the toxins, such that the Cry2Aa1 toxin interacts with receptors of species of Diptera, Hemiptera and Lepidoptera while Cry18Aa1 interacts with receptors of Coleoptera [21]. Despite these differences, cluster II also showed conserved thermodynamic behavior among its genes. The regions that encode domains I and III behaved similarly to *cry11Ba1* and *cry11Bb1*, but they all showed consistent thermodynamic behavior in the region encoding domain I, which had the greatest tendency to form secondary structures (Fig. 4). The thermodynamic behavior found for clusters I and II would be related to the conformation of conserved sequence blocks and to the protein structures of domains II and III of Cry toxins, whose evolutionary differentiation is determined by positive selection to interact with different receptors located in the insect midgut [26]. Similarly, the thermodynamic behavior observed in the region that encodes domain I may be related to preserving the functionality of the domain responsible for pore formation and oligomerization [26]. This idea is consistent with the experimental shuffling assays with *cry11* genes, where the reassembled products of *cry11Aa* showed only deletions of 3 to 90 amino acids in the amino-terminal region of the toxin. Interestingly, none of the assembled variables comprised the α4 and 5 helices involved in pore formation, and all of them showed toxic activity [Unpublished observations, Florez AM, Suarez-Barrera MO, Morales GM, Rivera KV, Orduz S, Ochoa R, Guerra D, Muskus C].

Genes phylogenetically distant from clusters I and II, such as the *cry1Aa* and *cry1Ab* genes in cluster III and the *cry30Aa* and *cry30Ca* genes in cluster IV, were used to determine whether the same behavior was maintained in other toxins. These groups were subjected to the same analysis. Cluster III showed a behavior that differed from that of clusters I, II and IV but was similar in the region encoding domain I. The region encoding domain I showed a lower tendency at the thermodynamic level to form secondary structures, and an inverted pattern was observed in both genes relative to the regions encoding domains II and III. However, in the Cry30 holotype, the pattern is maintained between genes but is different from the patterns observed in clusters I, II and III under shuffling conditions (Fig. 4). The results obtained for Cry1 contrast with the results obtained for Cry11; the most relevant difference is the low spontaneity in region I, suggesting that this region, which encodes the first domain of the Cry1Aa1 and Cry1Ab1 proteins, is the most thermodynamically stable and is in principle less prone to the formation of secondary structures. Under the simulated conditions of DNA shuffling used in this study, such stability might favor recombination and hence greater genetic variability. However, among experimental studies with Cry1 toxins that used DNA shuffling and combined methodologies and that showed preferences for modifications of domain III that were associated with increased toxic activity [27], only one study mentions previous fragmentation of the DNA, which resulted in few clones with activity and none with increased activity [30]. According to the authors, this result occurred because the toxins are not tolerant to the interchange of domains or to mutations in the conserved domains, where domain interchange is likely to occur [30].

The conserved behavior between the regions of the *cry30Aa1* and *cry30Ca1* genes (Fig. 4) is related to the identity of 78.1% between them. These genes showed a conserved thermodynamic pattern in all domains, and according to the structural analysis performed for Cry30Ca2, they share structural topology with Cry4Ba, with larger differences in domain II [31]. In any case, structural studies and studies of lethality of these toxins that allow comparisons among the results remain lacking. As this study is the first of its type on Cry toxins, there are no specific data that allow comparisons of the thermodynamic results found in our study with those of other studies in terms of genetic variability in regions that encode the protein domains during recombination events. However, our findings demonstrate the complexity of DNA shuffling at the experimental level in Cry toxins and highlight the need to design *in silico* models that allow the study of thermodynamic variables while improving the efficiency of assemblies that encode functional proteins.

## Conclusions

The observed thermodynamic variations allowed us to define a conserved pattern of domain behavior in analyses of different *cry* gene clusters. The conserved behavior was described in terms of thermodynamic spontaneity in ∆G values; it was used as a measurement criterion because $\overline{\varDelta G}\left(-\right)$ was the most representative estimator of the data with statistically significant differences. The most representative simulation scenario was LE-MA, as it showed significant *f-ratio* differences for all estimators, which were used as criteria to refine the *in silico* strategy for application to other groups in the *cry* gene family.

The thermodynamic behavior conserved among domains is associated with their phylogenetic closeness, suggesting that the observed patterns meet intrinsic conditions of the gene sequences, which are evolutionarily conserved and behave differently under DNA shuffling conditions. This behavior allows certain domains to be preferred because they assemble more efficiently under shuffling conditions.

These findings, in terms of spontaneity in domains with conserved behavior patterns, are useful for refining *in silico* models for DNA shuffling of *cry* genes using a new computational evaluation criterion for the selection of parental genes and for predicting, by qualitative analysis, the possible structural preferences of different variants to obtain gene assembly by computational biology.

## Abbreviations

LE-MA: Length - magnesium
LE-TE: Length - temperature
SANAFold: Statistical analysis of nucleic acid folding
TE-MA: Temperature - magnesium
